# Advanced Strategies of Salt Blocking in the Hypersaline Water

**DOI:** 10.1002/gch2.70124

**Published:** 2026-06-08

**Authors:** Aster Aberra Tessema, Tsegaye Deyou, Hirko Taye Debele, Fetene Fufa Bakare

**Affiliations:** ^1^ College of Natural and Computational Sciences Salale University Fiche Oromia Ethiopia; ^2^ Department of Advanced Materials Science and Engineering Center of Exellence Adama Science and Technology University Adama Ethiopia

**Keywords:** anti‐fouling and edge crystallization, hypersaline water, photothermal materials, solar energy

## Abstract

In the past decade, due to high consumption of freshwater and severe drought on the earth, human beings have experienced an unprecedented freshwater crisis. Solar‐driven desalination assisted by photothermal conversion materials is a new technology that mitigates the shortage of clean water in a green way. In this review, six advanced strategies have been discussed, which are the hydrophobic effect, unidirectional brine transport, zwitterionic effect, edge crystallization, siphon capillary effect, and dissolution‐based salt migration to avoid salt scaling in hypersaline water systems. We greatly expanded the mechanistic understanding of these approaches by studying major phenomena such as wettability, capillary flow, concentration‐gradient‐driven back‐diffusion, Marangoni flow, Donnan exclusion, and hydration‐layer effects. Finally, we proposed future research directions from a scientific perspective to direct the field and encourage practical implementation. In particular, we propose to develop multiscale porous structures combining edge crystallization with unidirectional transport for balanced water supply, evaporation, and salt removal. These systems could extract valuable mineral resources and freshwater from seawater simultaneously.

## Graphical abstract

Advanced strategies of salt blocking in hypersaline water*—*including hydrophobic, unidirectional brine transportation, dissolution migration, edge crystallization, siphon capillary effect, and zwitterionic effect.

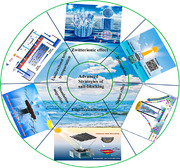

## 1 Introduction

The scarcity of freshwater is the most severe global problem that perplexes modern society [1]. It was estimated that 3.5 million people in underdeveloped countries could be plagued by a shortage of portable water by 2025 [2, 3]. Generally, sea water accounts for 96.5% of the total water capacity on Earth [4]. However, the salinity of seawater is so far from drinking water that humans cannot tolerate it [5]. Thus, seawater desalination is a promising technology to alleviate the crisis of fresh water [6, 7]. Unfortunately, commercial desalination technology has evolved significantly over the past century. Historically, large‐scale desalination began in the early 20th century with the thermal distillation method dominant until the 1970s [8, 9]. The main principle was to heat seawater to produce vapor, then condense it into fresh water. The salts and impurities are left behind in the brine solution. The thermal distillation method includes multi‐stage flash (MSF) and multiple‐effect distillation (MED). The main advantage of thermal distillation is generating high water purity, reliability, and long operating life with proper maintenance [10, 11]. Limitations include very high energy consumption, corrosion and scaling, high operational costs, large plant footprint, and high greenhouse gas emissions [12, 13, 14, 15] and the second commercial desalination process is reverse osmosis (RO), which was created in the middle of the 20th century and made available for purchase in the 1970s. RO is currently the most used desalination technique; its basic idea is to separate salts from water using high pressure and semi‐permeable membranes [8, 16, 17, 18]. The benefits of reverse osmosis (RO) include reduced energy consumption, cost‐effectiveness, large‐scale production, and a decreased environmental impact; nevertheless, its drawbacks include the need for feedwater pretreatment, membrane fouling, membrane replacement, and brine disposal concerns [19, 20, 21, 22], Moreover, when the salinity of brine concentration is over 7 wt.%, the life service of reverse osmosis membranes is dramatically stopped during desalination [23, 24, 25]. Therefore, the development of eco‐friendly and green energy to obtain fresh water by reducing carbon emissions is highly desirable [26].

Solar energy is renewable, clean, and free energy that is accessible almost everywhere in the world. It is an easy technology to implement and the most promising renewable source of energy in environmental remediation [27, 28, 29, 30]. Currently, solar steam generation is one of the most well‐known applications of solar energy, which also includes wastewater treatment and seawater desalination [31, 32, 33, 34]. Solar desalination is a cost‐effective, eco‐friendly, sustainable, and emerging technology that utilizes photothermal material to convert solar energy into thermal energy and harvests freshwater from sea or brackish water [35, 36, 37]. During solar desalination, the accumulation of salt is the bottleneck, which will block the water transportation channel and decrease light absorption, causing a dramatic decline in water evaporation efficiency. Besides severely influencing the life of the evaporator system. To overcome these issues, in the previous reports, the accumulation of salt crystals was removed by natural reflux and physical cleaning [38, 39], or by various post‐treatments methods, such process consumes manpower, time and material resources which may be increase the cost and reduce the output of water vapor. Additionally, photothermal conversion material is loss during washing and reduce the lifetime of evaporators. Therefore, solar evaporator with antifouling capacity has been urgently desired and remains challenge.

In solar desalination technology, photothermal conversion materials have gained extensive interest [40, 41, 42, 43]. These materials, which mainly include noble metals [34, 44, 45, 46], semiconductor [47, 48, 49, 50, 51, 52], carbon [53, 54, 55, 56] and polymer‐based materials [57, 58, 59, 60, 61] have been established to acquire excellent light‐to‐heat conversion efficiency during desalination.

**FIGURE 1:**
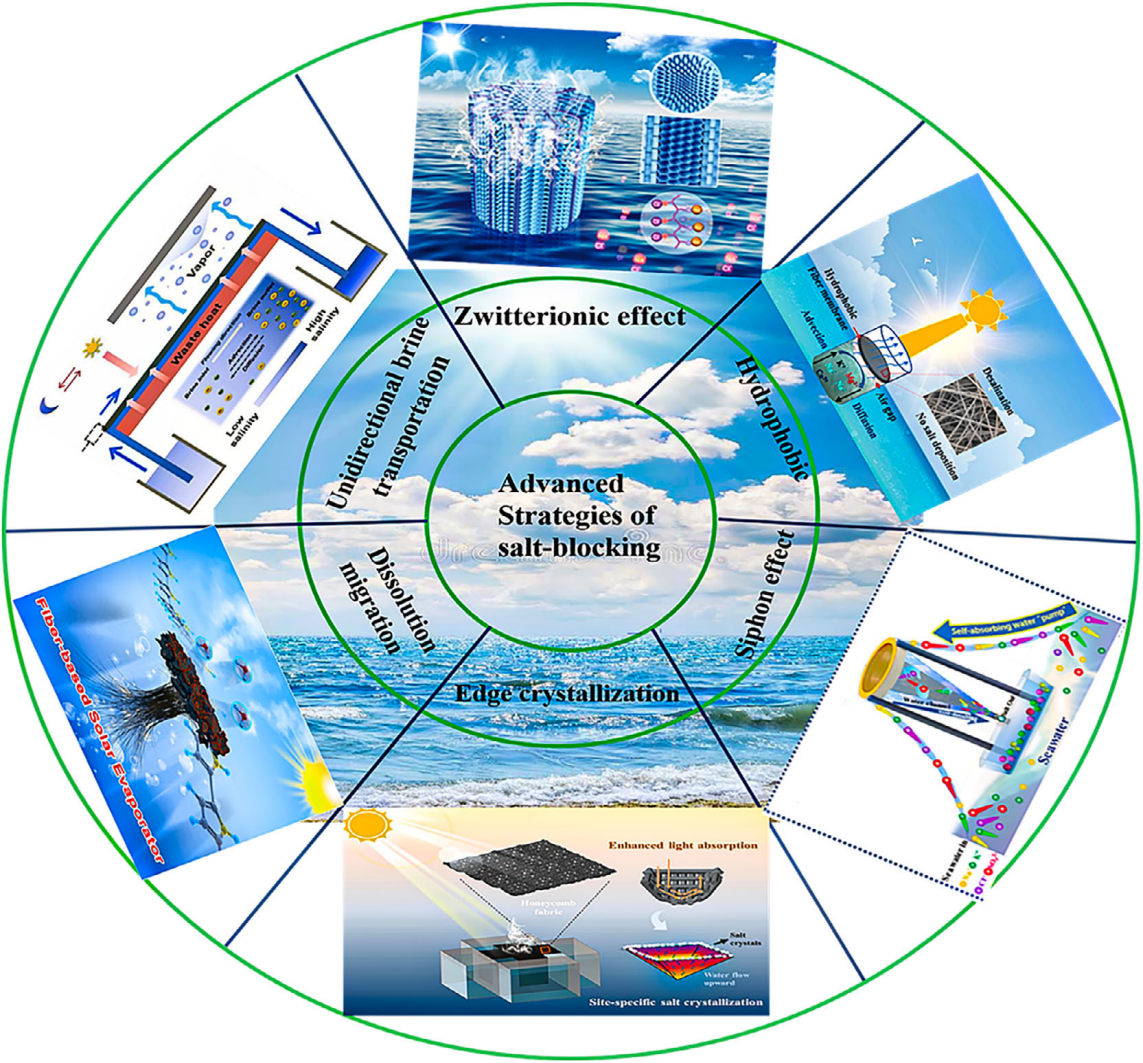
Schematic diagram of the classification of salt rejection strategies siphon effect, reproduced with permission [62]. Copyright 2022, Elsevier. edge crystallization, reproduced with permission [63]. Copyright 2022, Elsevier. hydrophobic effect, reproduced with permission [64]. Copyright 2021, Elsevier. Diffusion migration is reproduced with permission [65]. Copyright 2021, Elsevier. Zwitterionic effect is reproduced with permission [66]. Copyright 2022, Wiley‐VCH. Unidirectional brine transportation, reproduced with permission [67]. Copyright 2022, Elsevier.

Here, we review the structural design of a photothermal evaporator for salt‐blocking technology. First, traditional desalination technologies such as multiple‐effect distillation and reverse osmosis (RO) are discussed in the introduction. Then, the latest structural design of salt scaling prevention strategies is summarized as illustrated in Figure 1, In particular, our manuscript systematically organizes the field into six salt‐blocking strategies—hydrophobic effect, unidirectional brine transport, dissolution–migration, edge crystallization, siphon capillary effect, and zwitterion effect—which, to the best of our knowledge, have not been collectively classified or comparatively analyzed in prior reviews. Moreover, the effectiveness of the reported strategies in hypersaline solutions is summarized. And also, major challenges and future research perspectives are stated to provide insight for researchers. We hope this review will provide a road map for future research directions in desalination, specifically in hypersaline solutions.

## 2 Advanced Strategies for Salt Scaling Prevention

Solar desalination is a powerful technology to address the global portable water crisis. However, the accumulation of salt on photothermal conversion material during desalination blocks the incident sun light, consequently deteriorates the water evaporation rate, and finally limits the lifetime of the evaporator [68, 69]. To overcome these serious problems, many innovative techniques for salt scaling prevention have been developed [70]. Here, according to their working mechanisms, we classified them into six main strategies: hydrophobic effect, unidirectional brine transportation, zwitterionic effect, edge crystallization, siphon capillary effect, and migration of salts by dissolution. These strategies have been introduced as follows:

### 2.1 Hydrophobic Effect

**FIGURE 2:**
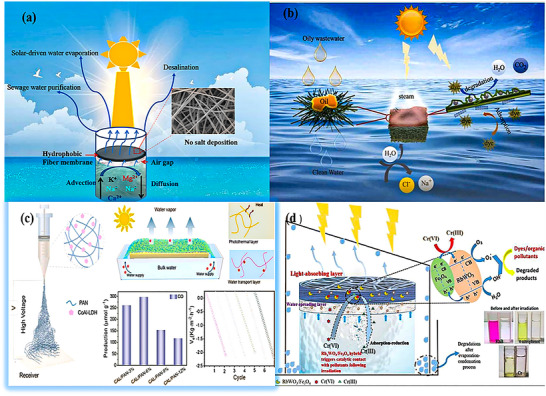
Illustrates the antifouling mechanism of hydrophobic fiber membranes, reproduced with permission [64]. Copyright 2021, Elsevier. (b) multifunctional cotton with polyaniline (PANI) and Ag NPs for oil, organic pollutants removal and solar–driven desalination, reproduced with permission [75]. Copyright 2022, Elsevier (c) a bilayer membrane construction with a CAL/PAN substrate and a CNT top layer, reproduced with permission [85]. Copyright 2026, Elsevier (d). fiber membranes for solar desalination and photo catalysis of organic pollutants, reproduced with permission [49]. Copyright 2022, Elsevier.

The first and most straight forward strategy to prevent salt ion accumulation on photothermal material is to utilize a hydrophobic evaporator [71, 72, 73]. The working principle of hydrophobic is that the surface repels liquid water, preventing pore wetting; only vapor passes through the membranes, improving salt rejection and long‐term flux. For instance, Tessema et al., [64] have developed Cs_x_WO_3_@g‐C_3_N_4_/polyvinylidene fluoride hydrophobic fiber membrane by electrospinning for desalination of seawater. Due to the surface hydrophobicity of the fiber membrane, an air gap was formed between the water surface and the fiber membrane. The formation of this air gap plays a crucial role in allowing water vapor to penetrate through the membranes while pollutants, especially salt ions, are repealed by the hydrophobic surface of the membranes and diffuse back to bulk water as shown in Figure 2a. By using this fiber membrane, an average rate of water evaporation of 2.68 kg m^−2^ h^−1^ and a photothermal conversion efficiency of 95.4% were achieved without the accumulation of salts over 40 cycles and more than 99.9% of main salt ions were rejected. Zhu's group [74] also reported a water lily‐inspired hydrophobic hierarchical structure for solar desalination. In this design, a thin water layer was confined between the superhydrophobic absorber and the bottom. Afterward, salt diffuses back into the bulk water. The rate of water evaporation of 1.27 kg m^−2^ h^−1^ over 18 days has been achieved. The advantage of using a hydrophobic evaporator is that it directly blocks salt crystallization and, due to its nonwetting property, exhibits great recyclability, self‐cleaning, and anti‐salt fouling abilities. Li et al. [75] also demonstrate multifunctional cotton with *PANI‐Ag NPs* that simultaneously shows oil resistance, organic pollutant removal, and a high water evaporation rate. The designed material achieved a water evaporation rate of 1.37 kg m^−^^2^ h^−^^1^ and a photothermal conversion efficiency of 84.7% under one‐sun illumination. Visible‐light catalytic degradation and adsorption thoroughly removed various organic pollutants in the water source, achieving efficiencies of 99.3% and 97%, respectively. It also possessed excellent superoleophobicity and repelled oil contaminants and organic pollutants in water as illustrated in Figure 2b. For the purpose of purifying oily seawater, Zhu et al. [76] developed composite membranes made of nanotubes and cellulose. Under 1.0 sun, the membrane could attain water evaporation rates and efficiencies of 1.58 kg m^−^^2^ h^−^^1^ and 90.86%, respectively. The proposed membrane demonstrated superhydrophobicity under oil and superoleophobicity underwater, supporting the integration of oily seawater purification. The clean water obtained by this composite membrane was in accordance with drinking water requirements.

In addition to hydrophobic and superoleophobic membrane technologies, advanced nanofiber‐based membranes have emerged as highly promising candidates for solar desalination [77]. Recent developments emphasize hierarchical and multifunctional nanofiber architectures, which combine interlaced porous networks with tunable pore size distributions and ultra‐high porosity [78]. These features significantly enhance broadband light absorption through multiple internal reflections and enable efficient vapor transport via interconnected channels, while minimizing conductive and convective heat losses [79, 80]. Moreover, incorporating photothermal nanomaterials—such as plasmonic nanoparticles, carbon‐based nanostructures, and semiconductors—into nanofiber matrices has significantly enhanced solar‐to‐thermal conversion efficiency [81]. The high specific surface area and tunable surface chemistry of these membranes promote efficient interfacial evaporation, while also improving reactivity and selectivity in separation processes [82, 83, 84]. In addition to solar desalination, they enable synergistic applications across a broader range of technologies. For instance, Xia et al. [85] fabricated a bilayer nanofibrous membrane via electrospinning, in which a photothermal PAN/CNT mat was deposited onto a substrate, as illustrated in Figure 2c. This layered architecture integrates efficient solar absorption and heat localization with a continuous capillary‐driven water supply, enabling synergistic effect. The incorporation of cobalt–aluminum layered double hydroxide (CoAl‐LDH) Nano sheets into the PAN matrix at the bottom layer enhances surface hydrophilicity and promotes water uptake, while carbon nanotubes (CNTs) impart strong broadband light absorption and excellent photothermal performance to the top layer. This hierarchically engineered bilayer membrane enables the simultaneous realization of photothermal water evaporation and photocatalytic CO_2_ reduction within a single solar‐driven platform. The membrane establishes a stable, capillary‐regulated gas–liquid–solid microenvironment, which is crucial for supporting both evaporation and catalytic processes. Consequently, the system achieves a high water evaporation rate of 2.5 kg m^−^^2^ h^−^^1^, along with an effective CO_2_‐to‐CO conversion rate of 287.6 µmol m^−^^2^ h^−^^1^.

In similar ways. My lab colleagues have developed a multifunctional RbxWO_3_@Fe_3_O_4_ composite immobilized within recycled triacetate cellulose (rTAC), which serves as the matrix, with polyethylene terephthalate (PET) used as the supporting substrate via electrospinning. The resulting bilayer fiber membrane consists of a hydrophobic rTAC top layer embedded with the functional composite and a hydrophilic nonwoven PET bottom layer. This engineered membrane exhibits a high water evaporation efficiency of 89% and achieves 99.9% salt ion rejection. In addition, the membrane demonstrates photocatalytic activity, effectively converting harmful pollutants in bulk water into harmless products, as illustrated in Figure 2d [49]. Wang et al. [86] also developed a dual‐scale porous photothermal/photocatalytic flexible nanofiber membrane for solar‐driven interfacial evaporation (SIE) and in situ degradation of volatile organic compounds (VOCs). The mesoporous, oxygen‐vacancy‐rich TiO_2__−__x_ nanofiber membrane (m‐TiO_2_‐NFM) provided abundant photocatalytic active sites, while the microporous network formed by intertwined fibers prolonged the residence time of VOCs and enhanced light absorption. As a result, when the m‐TiO_2_‐NFM was used to treat a 10 mg L^−^^1^ phenol solution, the phenol concentration in the condensed water decreased to 0.45 mg L^−^^1^, which is significantly lower than the 10.5 mg L^−^^1^. Simultaneously it was also applied to purify real river water under natural solar irradiation, and the resulting distilled water met China's drinking water standards.

The other structural design under hydrophobic systems is the Janus membrane, which has been discussed in detail in our previously published review paper [87]. Various approaches to hydrophobic desalination are summarized in Table 1.

**TABLE 1:** Summarizes different approaches to hydrophobic desalination.

Approaches	Design principles	Advantages	Refs.
Hydrophobic surface coating	laying the photothermal layer on substrates such as foams, textured copper, aerogels, and graphene oxide	Simple & scalable	[88, 89]
Janus structure	The top face is hydrophobic photothermal layer and bottom layer hydrophilic wick	Stable water supply and anti‐wetting. Continuous operation	[90, 91, 92, 93, 94, 95]
Omniphobic membrane	Combine hydrophobic and hierarchical roughness	Reduces membrane fouling and wetting. Long service life	[96, 97, 98, 99]
Hybrid photothermal hydrophobic membrane	Adding photothermal inside hydrophobic membrane matrices	Enhanced water flux	[64, 100]

Among the designs, Janus architecture is recommended due to the stable water supply and anti‐wetting.

### 2.2 Unidirectional Brine Transportation

**FIGURE 3:**
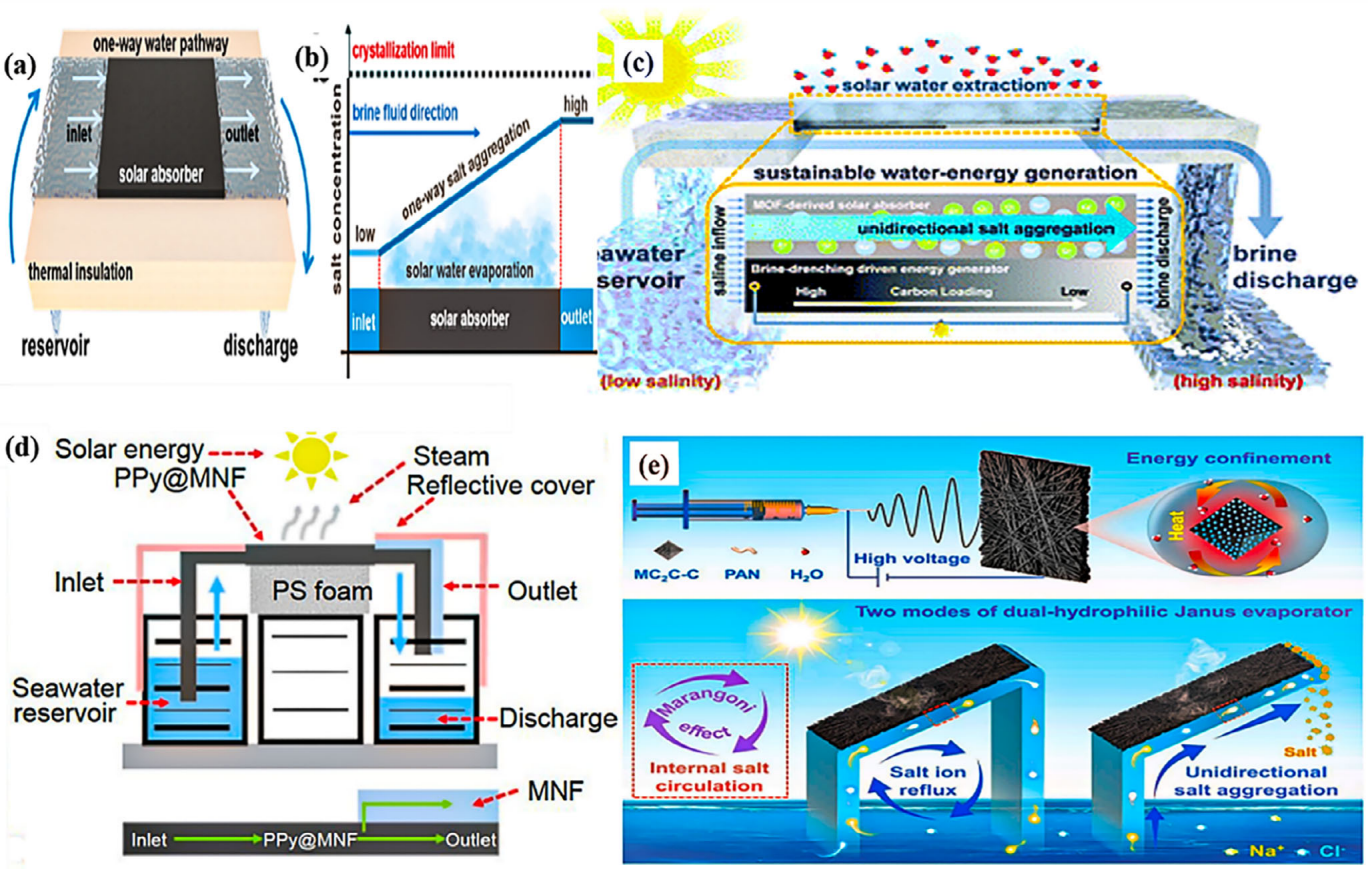
(a) Schematic illustration of photothermal structure for salt‐rejecting from one‐way saline transportation and (b) working mechanisms and salt‐rejection kinetics, reproduced with permission [104]. Copyright 2020, American Chemical Society. (c) Photothermal structural design for unidirectional brine transportation and drenching, reproduced with permission [105]. Copyright 2020, Royal Society of Chemistry. (d) One‐way brine transportation solar desalination device, reproduced with permission [106]. Copyright 2022, Springer, (e) structure and mechanism of unidirectional brine transportation, reproduced with permission [107]. Copyright 2023, Elsevier.

Another promising strategy for preventing the accumulation of salt is unidirectional brine transportation, in which the controlled one‐way movement of concentrated brine through designed channels, or membranes, minimizes salt accumulation and backflow [101, 102]. It is designed with water inlets as well as outlets. In the meantime, excessive amounts of salt ions flow out of the evaporator under the force of unidirectional water flow. The unidirectional brine transportation carried salt ions inside the photothermal material and discharged them outside via the outlet. This strategy avoids salt crystallization, which slows down the water evaporation rate [103]. For instance, Zhang et al. [104] proposed unidirectional brine transportation. As illustrated in Figure 3a, the structure consists three components, which are a brine water inlet, a hydrophilic solar evaporator, and outlet for salt discharge. During solar evaporation, the seawater feed into the solar evaporator through the inlet, in the time being the salt crystal is discharged via the outlet. As shown in Figure 3b, the concentration of salts is higher at the outlet than in the inlet zone, and water vapor is regularly generated from one‐way saline transportation. During solar desalination, an average water evaporation rate of 1.56 kg m^−2^h^−1^ followed by a solar water evaporation efficiency of 92%, was achieved under 1 illumination. The system was stable for over 100 h without salt crystallization. Zhang et al. [105] also presented MOF‐derived mesoporous carbon for unidirectional brine transportation. During the desalination process, the brine water coming from the inlet is low in salinity. However, the concentration of salt is rising along the direction of flow as a result of water loss. After the brine water totally traverses over the photothermal layer and arrives at the outlet, its concentration is much higher than the inlet salinity (Figure 3c). By this system, a rate of water evaporation of 1.6 kg m^−2^ h^−1^ was achieved under 1 sun irradiation. Sun et al. [106] designed high‐efficiency all weather evaporator by coupling photo‐electro thermal in single system (Figure 3d). In this design, the photothermal layer is prepared by deposition of polypyrrole on nonwoven fabric, while the electro‐thermal layer is fabricated by coating silicone resin and silver NPs on polypropylene fabric. The system achieved a rate of water evaporation of 2.61 kg m^−2^ h^−1^ under 1 s sun illumination. The one‐way brine transportation effectively prevented the accumulation of salt crystal during 24 h continuous desalination. Similarly, Li et al. [107] designed a dual‐hydrophilic inclined solar desalination evaporator, as shown in Figure 3e. The unidirectional brine transportation is because the inclined structure establishes a salinity gradient, and the highly hydraulic conductivity difference effectively prevents the Marangoni effect during solar evaporation. As a result, the constructed evaporator steadily worked for 200 h in a 10 wt.% saline solution without salt accumulation. The advantage of this strategy is the effective inhibition of salt crystallizations. Limitation: it needs an external force for brine transportation [2, 108, 109, 110]. Table 2 summarizes different approaches of unidirectional brine transportation.

**TABLE 2:** Summarizes different approaches to unidirectional brine transportation.

Approaches	Mechanism	Advantages	Refs.
Capillary pressure gradient	Arising from pore size variation drives one‐way transport of brine or water.	Minimizes salt crystallization and fouling. Energy efficiency. Long‐term anti‐fouling.	[111, 112, 113, 114, 115]
Wettability gradient	Water spontaneously directionally migrates in one direction, and brine can be rejected. This phenomenon occurs on a surface that exhibits a gradient from hydrophilic to hydrophobic.	Continuous operation. Long‐term stability. Drives spontaneous.	[112, 116, 117, 118]
Osmotic pressure gradient	Osmotic flow is caused by varying salt concentrations across semi‐permeable barriers.	Driven spontaneous Works synergistically with other gradients.	[112, 119, 120, 121]
Marangoni flow	Surface‐tension gradient caused by temperature gradient drives liquid in one direction	Improving evaporation efficiency. Energy‐efficient driving force. Simple and scalable.	[115, 122, 123, 124]

### 2.3 Dissolution Migration

**FIGURE 4:**
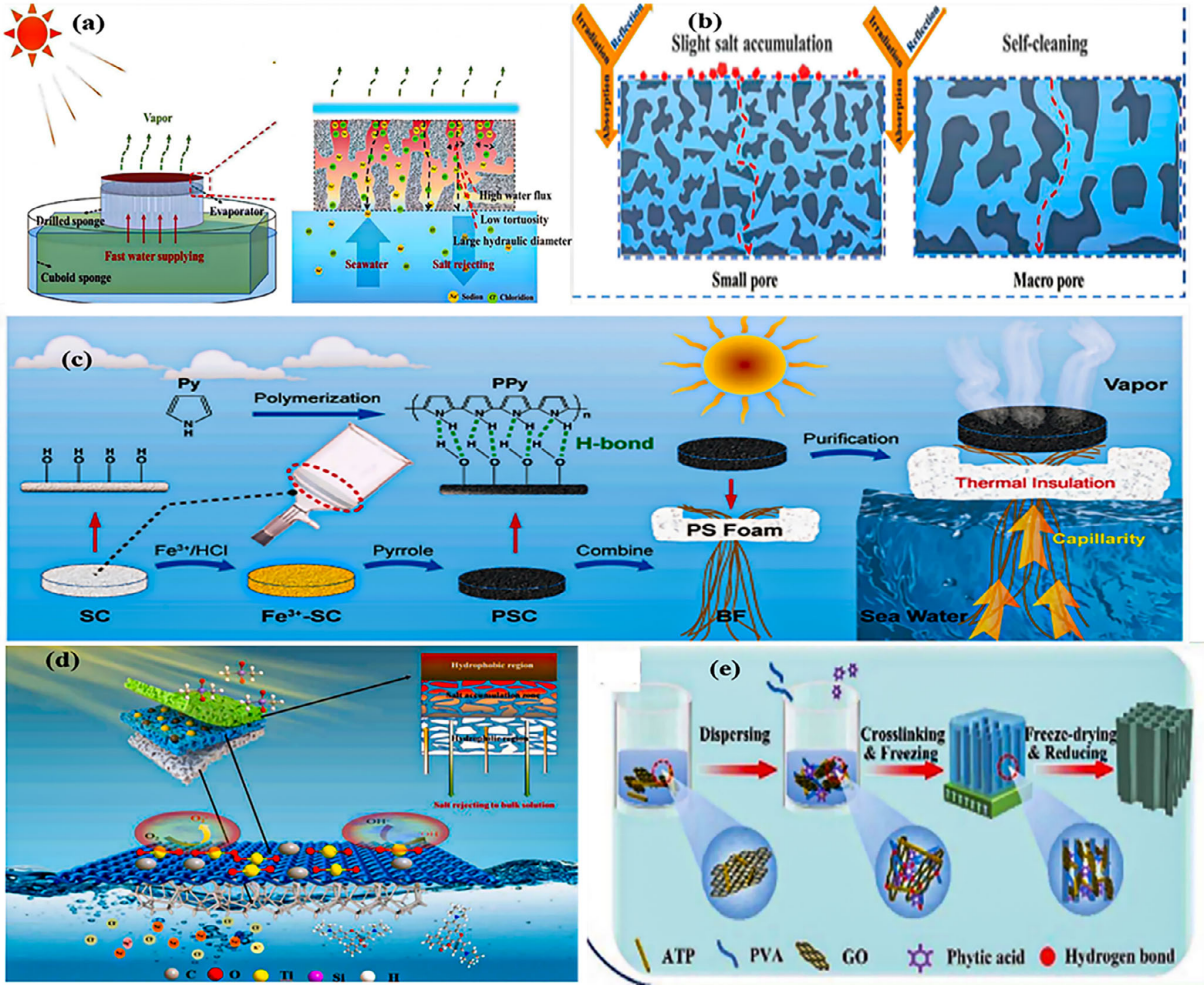
(a) Schematic illustration of solar desalination. The right diagram expresses the diffusion process of brine in the evaporator, which has large pores. (b) The influence of pore size on salt crystallization, reproduced with permission [1]. Copyright 2021, Elsevier, (c) illustrates the synthesis procedure of functionalized sand core and basalt fiber as solar desalination devices, reproduced with permission [65]. Copyright 2021, Elsevier, (d) The three layers of Janus membrane, designed with a hydrophobic salt resistance layer, a photothermal layer from the top, and a photocatalysis layer from the bottom, are reproduced with permission [130]. Copyright 2021, Elsevier, (e) schematic synthesis procedures of attapulgite‐aerogel salt rejection for dissolution, reproduced with permission [131]. Copyright 2021, Elsevier.

Accompanied by salt accumulation on the surface of the evaporator, salt ions also spontaneously migrate back into the bulk water as a result of the concentration gradient [34, 72, 125, 126]. According to Fick's law, if the rate of migration of salt ions is greater than or equal to the rate of precipitation on the evaporative surface, the system is expected to attain continuous antifouling [1]. Thus, improving the rate of migration of salt ions via porous evaporators is critical [127, 128, 129]. For instance, Huang et al., [1] proposed a salt‐rejecting evaporator by depositing carbon blacks on the skeleton of polystyrene or lignocellulose, as presented in Figure 4a. The evaporator continuously rejects salt due to its high porosity (70.4%) and large pore size, which ranges from 150 to 300 µm. The high porosity supports the evaporator's ability to absorb plenty of water via water channels, facilitating fast salt exchange with seawater. As reported in Figure 4b large‐pore evaporator, shows excellent salt‐rejecting ability compared with small‐pore size due to two reasons, the first reason is, the large‐pore size evaporator has more hydraulic diameter to hold high amount of water in a single pore, hence can successfully limit salt ions crystallization. The other reason is that it has a lower tortuosity, which decreases the migration pathway of salt ions, further encouraging the diffusion of salt back into seawater. By using this system, 85.5% water evaporation efficiency was achieved in a high salinity (15%) environment.

Zhang's group [65] developed a novel indirect‐contact evaporator by modifying polypyrrole with a sand core (PSC) and basalt fiber, as demonstrated in Figure 4c. The basalt fiber is working as a water delivery channel to PSC and avoiding direct contact of PSC with bulk seawater, dramatically reducing heat loss during steam generation. While the porous structure of PSC is used to permit all the incident light to be scattered within the evaporator, Moreover, water transported via macropores dissolves the crystalized salt and returns it to seawater along basalt fiber to achieve self‐cleaning. This evaporator achieves 85.9% water evaporation efficiency under 1 sun irradiation with good self‐cleaning capability. Zhang et al. [130] also proposed three‐layer evaporator Figure 4d by dip‐spray coating process, the first layer is hydrophilic melamine sponges (MS) used as matrix, the second layer is photothermal conversion material Chinese ink/TiO_2_ while the top is thin layer Poly (dimethyl siloxane) (PDMS) sprayed on Chinese ink/TiO_2_@MS to endowing the upper layer with resist oxidation and hydrophobicity. Furthermore, the PDMS act as “glue” to integrate TiO_2_ and Chinese ink with the melamine sponges. Additionally, it prevents the photothermal material from dispersing into the water. During desalination salt ions crystallization is appeared in hydrophilic layer which quickly diffused back to bulk seawater via pores structure. As they reported two forces acting on crystalline salts, the first one is during sun light the negatively induced pressure attracts salt crystals in the upward direction. Consequently, salt ion nucleation and crystallization occur only in the hydrophilic region. When the light is off, gravity plays the main role and the upward attraction force is disappeared, resulting in the washing away of crystalline salt that exist in the hydrophilic region. Due to this the salt crystal can easily return back to the bulk seawater.

Mu et al. [131] also introduced attapulgite‐based aerogels which is prepared by freeze‐drying (Figure 4e). The as prepared aerogel has a vertical channel which provides pathways for water circulation and resisting salt crystallizations at high brine concentration. The water evaporation rate of this aerogel is ranging from 1.3 to 1.5 kg m^−2^ h^−1^ even at high‐salinity (20%) environments.

The advantage of this strategy is high concentrated salt tolerance. Limitation, it needs additional pathway for salt ions diffusion, unavoidable heat dissipation, the salt ion may be crystalized inside the evaporator which leads to decline the water evaporation performance, and also the valuable mineral cannot be collected by this strategy.

### 2.4 Edge Crystallization

**FIGURE 5:**
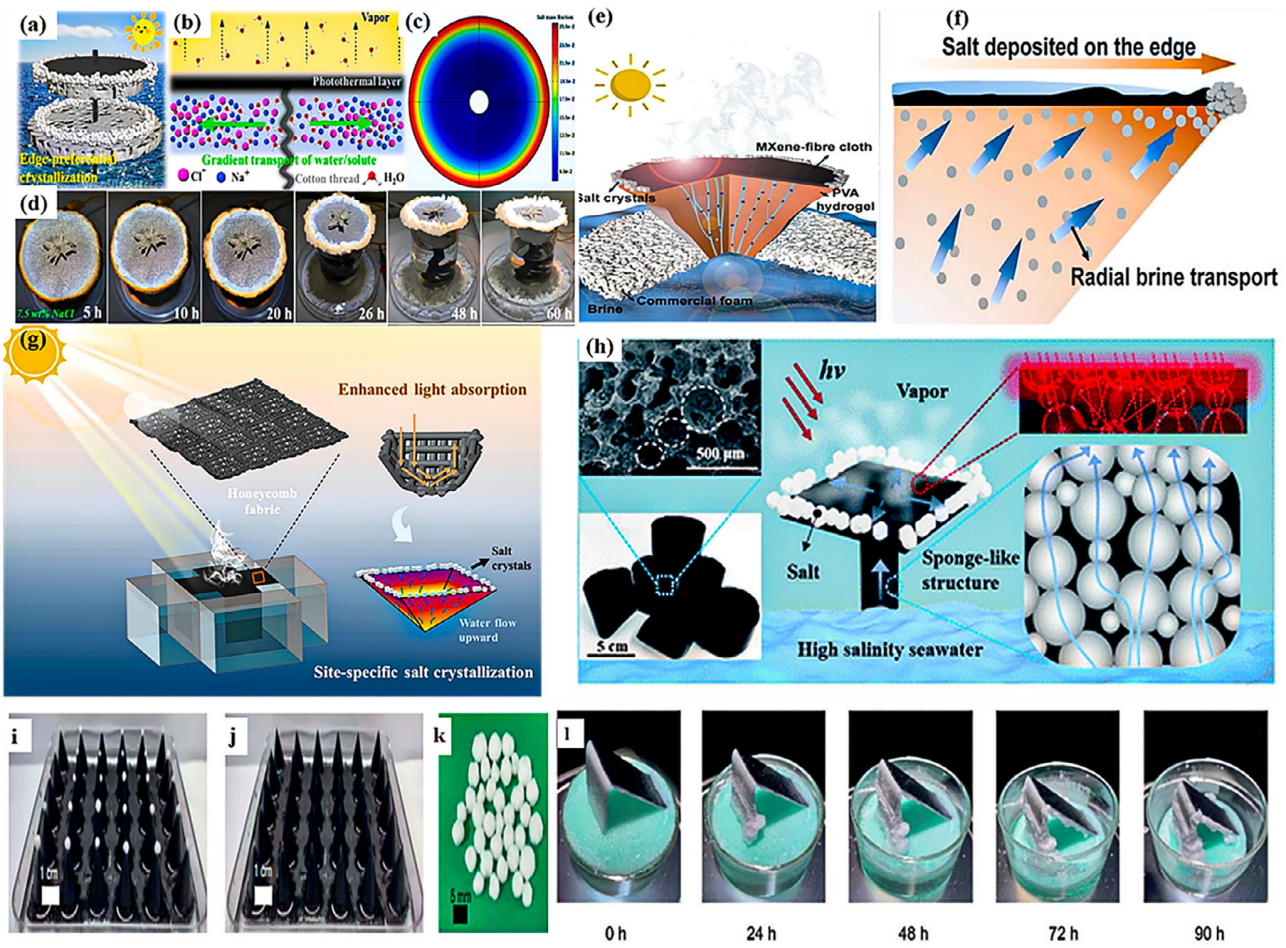
(a) Schematic illustration of solar desalination; (b) transport of salt ions in the evaporator, (c) simulation of the salt concentration gradient on the evaporator prophesied by computational fluid dynamics. (d) Digital photographs of preferential salt crystallization during solar desalination, reproduced with permission [134]. Copyright 2021, Elsevier, (e) Schematic illustration of solar desalination using conical frustum PVA, MXene‐fiber cloth, and commercial foam. (f) Edge salt crystallization as a result of radial brine transportation, reproduced with permission [135]. Copyright 2021, Wiley, (g) The schematic illustration of solar desalination and edge salt crystallization in honeycomb fabric, reproduced with permission [63]. Copyright 2021, (h) a biomass‐based hydrogel evaporator with salts crystalized at the edges of the evaporator, reproduced with permission [136]. Copyright 2020, Royal Society of Chemistry, (i), Optical picture of arrayed evaporators, and (j) after removing the crystallized salt. (k) Optical picture of the detached salt from arrayed evaporator, reproduced with permission [137] 2020 Copyright, Springer Nature, (n) Photographic image of the salt crystallizer at the edge, reproduced with permission [139]. Copyright 2022, Royal Society of Chemistry.

Edge crystallization deliberately directs salt precipitation to particular peripheral points of an evaporator. While keeping the central evaporation area clean and productive. Surface salts nucleate at tips and edges where concentration and supersaturation rise; crystals are subsequently removed by gravity or purposeful peeling. In zero liquid discharge systems, this approach permits passive salt harvesting and continual freshwater production [41]. The mechanism of this strategy is that water transports salt ions by capillary force at the margin of the evaporator. As water vapor gradually escapes, concentrated salts are precipitated at the edge and finally removed from photothermal material by gravity‐assisted or simply scraped‐off salt crystals [132, 133]. Recently, Peng and co‐workers [134] reported textile‐based MOFs coated with tannic acid and molybdenum disulfide for solar desalination. As demonstrated in Figure 5a,b, when a 7.5% wt.% salt solution was transported from the bottom to the textile, due to the capillary effect, it ran from the center to the edge of the textile. In the meantime, water evaporation is ongoing, and when unevaporated salted solution is near the edge of the evaporator, the salinity exceeds its solubility limit. Resulting edge‐preferential crystallization. According to the fluid dynamics model and simulation illustrated in Figure 5c, the mass of salts is higher at the edge of the disc than in the center part. Additionally, as their finding indicates in Figure 5d, the salt predominantly crystallizes at the edge of the evaporator in 60 h of continuous operation. And due to the weak adhesion between the evaporator textile and salt crystals, salts automatically fall off the evaporator under gravity.

Li et al. [135] constructed a PVA‐based 3D hydrogel evaporator for solar desalination. As illustrated in Figure 5e, on the conical frustum evaporator, the salt crystal was detected only at the edge, while the majority of the surface remained clean. As their finding indicated, the conical frustum design synergistically facilitates both radial brine transportation as well as edge‐preferential crystallization, as seen from Figure 5f. This 3D hydrogel evaporator shows excellent salt‐resistant ability without salt deposition on the evaporative surface under 20 wt.% brine solution for continuous 24 h solar irradiation. Gao et al. [63] have presented rGO and chitosan‐coated honeycomb fabric for solar desalination. Due to the inverted pyramid shape, the light‐capturing capacity of the fabric is enhanced, and a temperature gradient was formed from bottom to top, which induced the Marangoni effect as shown in the insert of Figure 5g. The flow of water was accelerated upward. Thus, the salt crystals were deposited on top of the honeycomb fabric. By this design, a 2.02 kg m^−2^ h^−1^ water evaporation rate was achieved under one sun illumination. Similarly, Shao, Chen et al. [136] reported a biomass‐based hydrogel fabricated with a foaming‐polymerization approach for simultaneous salt harvesting and steam generation, as illustrated in Figure 5h. This evaporator achieved a rate of water evaporation of 1.77 kg m^−2^ h^−1^ in a 25 wt.% hypersaline environment and continuously operated for 712 h without deterioration. During desalination, the salt is crystallized preferentially at the edge of the evaporator.

Wu et al. [137] also designed a bio‐mimetic, 3D evaporator for solar desalination. Importantly, the individual cone of the evaporator forms the temperature gradient that induces the Marangoni effect, resulting in edge crystallization of salt on the apex of every cone. In this system, water is carried by thermocapillary force from the bottom to the apex of the evaporator to enhance water evaporation efficiency. During solar desalination, the rate of water evaporation was 2.24 kg m^−2^ h^−1^ with a water evaporation efficiency of 97.1%, achieved under a 25 wt.% brine solution. Because of the very small area coverage of solid salt, the evaporator maintained stable water evaporation performance, while the accumulated salt on the apex of the cone was easily removed and harvested, as demonstrated in Figure 5i–k.

Wang et al. also introduced [138] integrated salt crystallization and solar evaporation zones in a single system. The designed structure consists of two jointed sections, an inclined and a vertical slice, which served as solar evaporation zones as well as salt crystallization zones, respectively, as illustrated in Figure 5l. The inclined surface end is immersed in brine. Salt ions and water are transported to an inclined slice and then, by capillary force, to a vertical slice. Under solar irradiation, only the inclined slice received solar light to drive solar evaporation, while the vertical slice received no light. The accumulation of salts only occurred vertically. Hence, the salt ions crystallization could not affect the rate of water evaporation. Over 90 h, a steady rate of evaporation of 1.71 kg m^−2^ h^−1^ and localized crystallization were achieved. Moreover, Shi et al. [139] presented a 3D cup‐shaped solar evaporator that permitted salt crystallization on the outer surface of the evaporator, whereas the inner wall undergoes water evaporation and photothermal conversion. The cup controls the edge crystallization by taking advantage of the temperature and humidity difference between the outer and inner walls of the cup. Still, at the bottom of the cup, a very small amount of salt crystals appeared when the concentration of brine was greater than 20%. Nevertheless, it did not influence the rate of water evaporation; this system maintained the rate of water evaporation at 1.3 kg m^−2^ h^−1^ for 2 h.

Advantages of edge crystallization strategy: consistent ability to localize nucleation, excellent stability under hypersaline conditions compared to alternative strategies, and capacity to facilitate salt collection, reduce environmental impact, and enable resource recovery. Additionally, the approach is inherently scalable: leaf‐ or umbrella‐like modules can be readily arrayed, and 3D‐printed geometries simplify both prototyping and scale‐up deployment. limitation: low evaporation rate at the edge of the evaporator. Based on our review, edge crystallization is considered a promising and controllable strategy.

### 2.5 Siphon Capillary Effect

**FIGURE 6:**
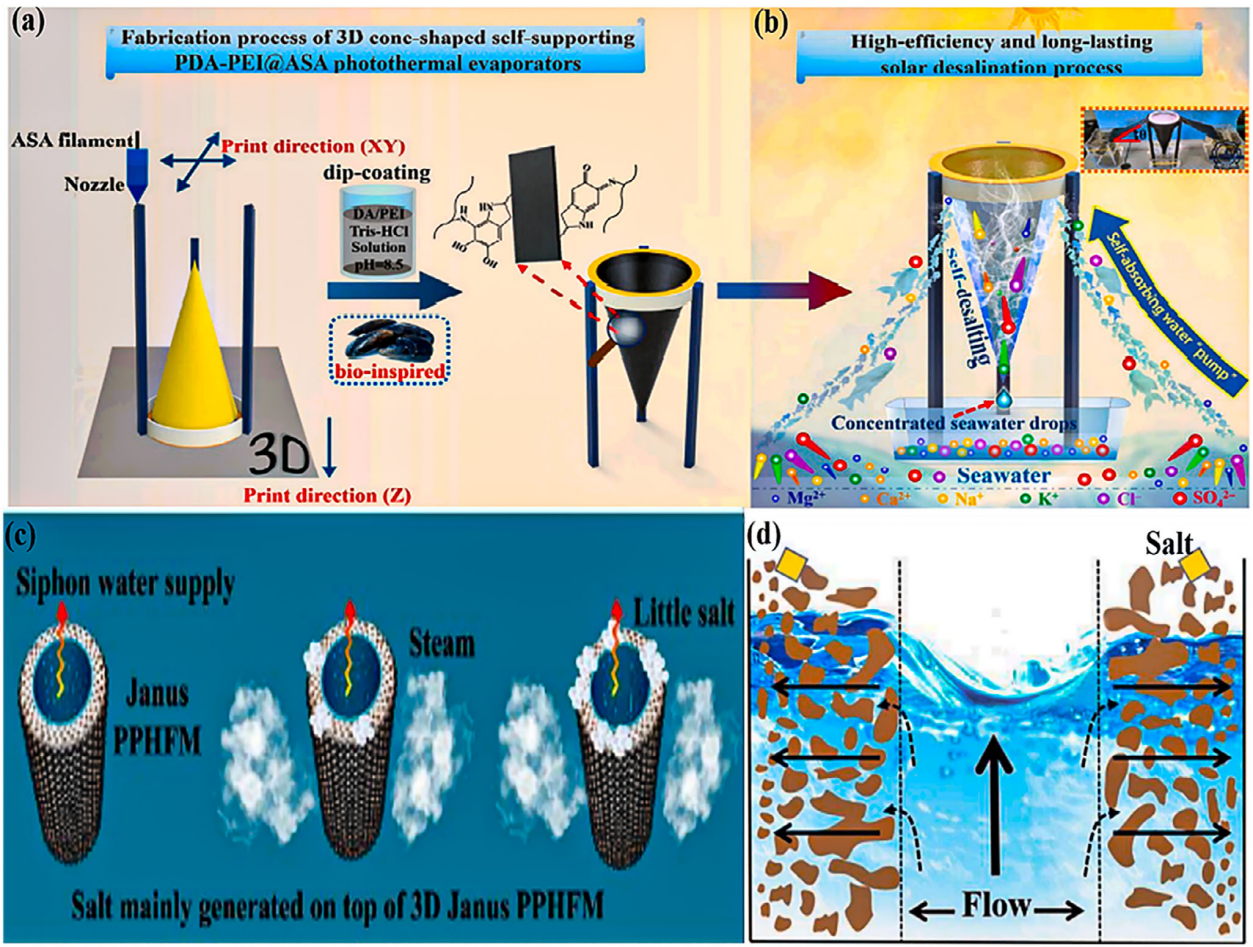
(a) Schematic illustration of the fabrication of cone‐shaped 3D photothermal evaporators, (b) cone‐shaped PDA‐PEI@ASA evaporators for application of seawater desalination and organic dye degradation, reproduced with permission [62]. Copyright 2022, Elsevier, (c,d) illustrate vertically aligned hollow fiber membranes for siphoning water supply and salt ions, rejection, reproduced with permission [140]. Copyright 2022, Elsevier.

As is well known, hydrophilic cellulose, which is found in stem trees, plays a vital role by transporting groundwater to the leaves by capillary‐driven siphon effect under the irradiation of solar light [140]. Inspired by this, Cao et al. [62] developed a novel self‐desalting siphon‐drop mode evaporator system through 3D printing and polymerization methods, as demonstrated in Figure 6a. The inner part of the 3D cone is decorated with Polydopamine and polyethyleneimine (PDA‐PEI) as solar light absorbers, while the outer part is with poly(acrylonitrile‐styrene‐acrylate) (ASA). As shown in Figure 6b, the seawater was continuously pumped to the top of the evaporator via the siphon effect to attain top‐down water diffusion on the evaporator surface. In this unique design, concentrated salts were dropped from the evaporator to the bottom before saturation. Additionally, the three legs style endows the evaporator with self‐supporting mode instead of traditional floating mode, which adequately controls the thermal loss toward bulk water. By this design, a 2.55 kg/m^2^h rate of water evaporation was achieved under a 20 wt.% hypersaline environment without accumulation of salt over 168 h. Wei et al. [140] also reported a vertically aligned 3D Janus hollow fiber membrane with asymmetric surface wettability, i.e., an inner 0° and an outer 90° water contact angle. The interior of hollow fiber was cross‐linked with PDA, KH550, and TiO_2_, whereas the outer was modified with PPy. This membrane shows excellent salt‐resistant capacity under 20 wt.% salinity conditions. As illustrated in Figure 6c,d, after running for two days, only a small amount of salt appeared on vertically aligned Janus HFMs. This evaporator also shows 229.1% photothermal conversion efficiency under 1.0 kW/m^2^, which exceeds the standard photothermal conversion limit. The calculated value of 229.1% in their study is attributed to the specific calculation method used, which includes not only the incident solar irradiation but also additional heat input from the surrounding environment. In particular, the tinfoil reflector and the system configuration allow (i) diffuse reflection of incident light, (ii) absorption of ambient thermal energy and (iii) convective and radiative heat feedback to the evaporation interface. This means that the measured evaporation rate is a combination of direct solar input and secondary energy inputs, leading to an apparent efficiency of over 100%. The advantage of this strategy is that it effectively suppresses salt accumulation in hypersaline environments with excellent photothermal conversion efficiency. Limitation: still, there is little salt crystallization at the hydrophobic and hydrophilic interfaces.

### 2.6 Zwitterionic Effect

**FIGURE 7:**
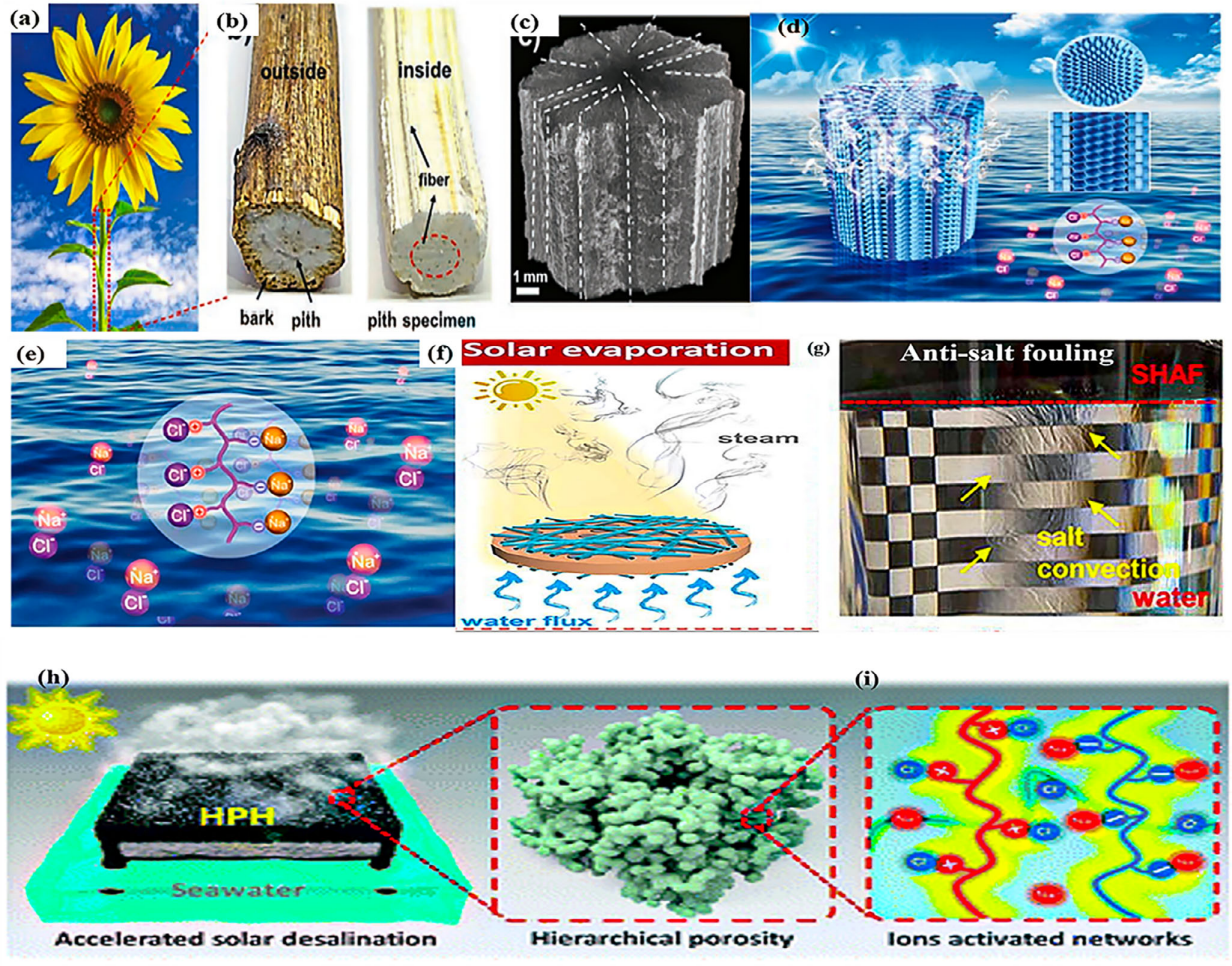
(a,b) Illustrate schematic images of the sunflower stalk and pith, respectively. (c) Demonstrates well‐aligned channels for the pith. (d,e) Illustrate the working principles of zwitterionic hydrogel coated sunflower pith and salt resistance mechanisms, respectively, reproduced with permission [66]. Copyright 2022, Wiley, (f) Solar desalination, the evaporator contacts water molecules and forms hydration layer because of the zwitterionic effect. (g) Illustrating the anti‐ salt fouling capabilities of the designed evaporator, reproduced with permission [148]. Copyright 2021, Elsevier. (h,i) Illustrate hierarchical porous polyion for solar desalination and salt‐resistance, reproduced with permission [149]. Copyright 2020, Royal Society of Chemistry.

Zwitterionic effect uses surfaces with both positive and negative charges that form hydration shells, repelling ions and preventing salt crystallization. Recently, zwitterionic material for desalination has been a research hotspot [141], mainly based on zwitterionic hydrogels. Zwitterionic hydrogels can interact with both water molecules and salt ions owing to the dense distribution of cationic and anionic charged groups [142, 143, 144, 145, 146]. This property makes them strong hydrators and salt‐tolerant. Zwitterionic hydrogels have unique properties such as excellent water absorption ability, electrostatic repulsion, and intrinsic photothermal conversion capacity [147]. For example, Su et al. [66] introduced a sunflower pith biomass solar evaporator. The sunflower stalk is composed of 90 wt.% outer fibers and 10 wt.% inner pitch, which were further modified with zwitterionic hydrogels and carbon black (Figure 7a,b). The hole shape of the pitch stalk is hexagonal with a regular configuration and honeycomb structure, as displayed in Figure 7c,d. This structure helps multiple reflections of light within the structure and rapid transportation of water to enhance the rate of water evaporation. By this design, a rate of water evaporation of 1.90 kg m^−2^ h^−1^ and an evaporation efficiency of 98.8% were achieved. After 50 h of continuous desalination, no accumulation of salt was observed in a 20% saline environment. This is due to the electrostatic interaction of zwitterionic groups on the hydrogel, which could capture oppositely charged Cl‐ and Na^+^ ions, which are very difficult for Cl‐ and Na^+^ ions to bind and precipitate on the surface of the evaporator, as illustrated in Figure 7e.

Wen et al. [148] also designed a superhydrophilic hierarchical zwitterionic coated polyurethane evaporator, as illustrated in Figure 7f. This design consists of polyurethane coated with zwitterionic hydrogel, polystyrene foam, which is used as a thermal insulator, and cotton swabs as a water delivery channel. Zwitterionic coatings adhere water molecules to make a hydration layer, which leads to constant water transport and fascinating anti‐fouling. Due to its integrated merits, this evaporator achieved a rate of water evaporation of 2.2 kg m^−2^h^−1^ followed by a 93.5% photothermal conversion efficiency. It also shows excellent anti‐salt fouling ability, as shown in Figure 7 g. Similarly, Zhu et al. [149] presented hierarchical zwitterionic hydrogels for desalination, as illustrated in Figure 7 h. The zwitterionic hydrogels continuously supplied water via hierarchical porous channels and hydrophilic networked hydrogels. The salt resistance of hierarchical zwitterionic hydrogels is due to rapid water replenishments, which speed up salt ion exchanges. Additionally, the zwitterionic cationic and anionic ions on the skeleton of the hydrogel were trapping oppositely charged ions of salts within the hydrogel network, which prevented the crystalization of salts on the evaporator (Figure 7i). This design exhibited a 2.79 kg m^−2^ h^−1^ rate of water evaporation, followed by 96.3% water evaporation efficiency under 3.5 wt.% salinity of sea water. Advantage: the crystalization of salts in evaporators is low due to the Donnan effect and size screening. Limitation: the charged hydrogels, in addition to blocking the salt ions, also impede the transport of water during desalination [141, 150].

All six strategies can be explained in terms of coupled interfacial and transport phenomena, such as capillarity (through wettability), Marangoni flow (through interfacial tension gradients), chemical potential gradients (through forward transport and back‐diffusion), Donnan exclusion (through electrostatic partitioning) and nanoscale hydration effects [151, 152, 153]. In fact, salt rejection in hypersaline solar desalination systems is interconnected rather than independent phenomena [154].

A number of processes are listed in Table 3, which are intrinsically coupled and not independent. For example, the salt transport pathways, liquid uptake and the local concentration gradients driving back‐diffusion are jointly determined by wettability and capillary flow [179, 180]. These concentration gradients may also result in Marangoni stresses, depending upon the surface tension gradients generated by temperature and salinity variation, the Marangoni stresses may enhance or suppress interfacial convection [181, 182, 183]. Alternatively, the formation of hydration layers is closely associated with the Donnan exclusion in charged or zwitterionic systems, which influences simultaneously the ion partitioning, interfacial diffusion resistance and the effective wettability of the system [184, 185].

**TABLE 3:** Summarizes the combined regulation of salt transport by wettability, capillary flow, concentration gradient induced back‐diffusion, Marangoni flow, Donnan exclusion, and hydration layer effects across six strategies.

Strategy	Wettability control	Capillary flow regulation	Back‐diffusion	Marangoni flow	Donnan exclusion	Hydration‐layer effects	dominant mechanism(s)	Refs.
Hydrophobic effect	Strongly hydrophobic surface limits liquid intrusion and salt nucleation	Suppressed capillary imbibition	Weak	Possible at liquid–vapor interface	—	Minimal	Wettability‐dominated salt rejection	[155, 156, 157, 158]
Unidirectional brine transport	Wettability gradient (hydrophilic to hydrophobic or vice versa)	Asymmetric capillary pumping enables one‐way flow	Reduced via continuous flushing	Can assist directional flow	—	Limited	Capillary‐driven directional transport	[133, 159, 160, 161, 162, 163]
Dissolution migration	Typically hydrophilic interface	Strong capillary supply of fresh water	Strong: dissolution and back‐diffusion removes ions	weak	—	Weak	Concentration‐gradient‐driven ion redistribution	[65, 130, 164, 165, 166]
Edge crystallization	Wettability gradient localizes evaporation zones	Capillary flow drives brine to edges	Reduced in central	Thermal gradients may induce Marangoni	—	Weak	Coupled capillary and evaporation localization	[133, 167, 168, 169, 170]
Siphon capillary effect	Hydrophilic supports the flow	Continuous capillary siphoning removes salt	Strong suppression of buildup	weak	—	Weak	Sustained capillary flushing	[171, 172]
Zwitterionic effect	Hydrophilic due to charged	Moderate capillary uptake	Reduced via ion exclusion	Minimal	Strong (electrostatic ion rejection)	Strong (hydration shells resist fouling)	Donnan exclusion and hydration barrier	[173, 174, 175, 176, 177, 178]

This review is focused on the effectiveness of different advanced strategies for salt scaling prevention in hypersaline water.Based on our findings and Table 4, we state the advantages and drawbacks of each strategy and their effectiveness when treating high‐concentration feeds as follows: (1). In the case of the hydrophobic evaporator, with a 10 wt.% brine solution, steady evaporation for 18 days was achieved. At the same time, due to its nonwetting property, water molecules are also being blocked with salt ions, which results in lower photothermal conversion efficiency. (2) unidirectional brine transportation countinously worked for 200 h in a 10 wt.% saline solution without salt accumulation. However, this strategy needs external force for brine transportation. (3). Some researchers reduce the accumulation of salt by constructing low and large tortuous channels in the evaporator to facilitate the dissolution migration of salt. With this design, up to 20 wt.% brine solution could be continuously desalinated for 100 h. However, the photothermal conversion efficiency dropped to 75%. This may be due to the accumulation of salts in the porous channel and, along with mass transfer, an invitable amount of heat also transfers to the bulk solution. (4). In the siphon effect strategy, a light‐absorbing and solar desalination zone is spatially isolated; it attained long‐term anti‐salt fouling (168 h for 20 wt.% NaCl) with a high rate of water evaporation of 2.55 kg m^−2^ h^−1^. However, there is little salt crystallization at the hydrophobic and hydrophilic interfaces. (5) Using the zwitterionic effect of hydrogel to prevent salt scaling on the evaporator is a novel strategy. With this approach, for 2–50 h, continuous desalination is possible at 20 wt.% in a saline environment. However, in a more concentrated salt solution, this approach is limited by the low osmotic pressure of zwitterionic hydrogels. In addition to salt ions rejection, zwitterionics also impede the transport of water during desalination. Which may lead to lower photothermal conversion efficiency. (6) edge crystallization strategy is working in a hypersaline water environment at 25 wt.% for 712 h in continuous desalination with a high 1.77 kg m^−2^ h^−1^ water evaporation rate. This can be achieved with an elaborately designed evaporator that leads the orientation of salt accumulation at the edge while keeping the active evaporation area away from saltfouling. Thus, the edge preferential crystallization strategy edge crystallization is considered a promising and controllable strategy under hypersaline seawater.

**TABLE 4:** Summaizing different stategies of salt blocking.

Salt blocking strategies	Evaporator	Concentration of salts (wt.%)	Evaporation efficiency (%)	Evaporation rate kg m^−2^ h^−1^	Stability	Power Density kW m^−2^	Refs.
Hydrophobic evaporator	Water Lily Structure	10	80.0	1.28	18 days	1	[74]
	Cs_x_WO_3_@g‐C_3_N_4_/PVDF fiber membranes	3.5	95.4	2.70	40h	2	[64]
	MXene membrane	3.5	71.0	1.31	200h	1	[186]
	MoO_3_x‐QDs membrane	3.5	62.0	4.95		5	[187]
	Cu /N‐doped‐graphene/PVDF	3.5	82.0	0.63	10h	1	[188]
	Black Titania/ PVDF	3.5	70.9		10h	1	[189]
	CNTs@PVP	3.5	91.1	1.40	40h	1	[190]
	Poly(1,3,5‐hexahydro‐1,3,5‐triazine) foam	3.5	80.0	1.17	100h	1	[58]
Uniderctional brine transportation	(PANi)/cellulose paper	10	92.0	1.50	100h	1	[104]
	MOF derivative mesoporous carbon	3.5		1.60	25h	1	[105]
	pyy@non‐woven fabric	15		2.61	24h	1	[106]
	Mo_2_C‐C@PAN	10	83.3	1.81	200h	1	[107]
	MoS_2_@graphene	3.5		3.20	10	0.9	[191]
Dissolution Migration	Carbon blacks/ polystyrene/lignocellulose	15	85.5	1.90	12h	1.5	[1]
	Chinese ink/TiO_2_@MS	3.5	93.5	1.49	6h	1	[130]
	Polypyrrole/ sand core/basalt fiber		85.9	1.26	20h	1	[65]
	Carbonized Wood	20	75.0	1.04	100h	1	[192]
	Flame‐treated wood aerogel	3.5	83.4	1.40	7days	1	[193]
	PPy@PEI@A‐CNF aerogel	10	94.6	1.66	48h	1	[194]
	Au−rGO/wood	3.5	90.1		120h	1	[195]
Edge Crystallization	3D Hydrogel	10 to 25		2.07	7 day (56 h)	1	[135]
	Honeycomb‐ fabric	20%	95.4	1.97	8	1	[63]
	TA‐MoS_2_/ UIO‐66‐NH_2_	7.5		1.36	60h	1	[134]
	CNTs/ Filter paper	3.5	81.2	1.42	600h	1	[196]
	3D Cup evaporator	20	96.7	1.30	120 h	1	[139]
	Bird beak mimetic evaporator	25	96.0	2.60	10 d	1	[137]
	Carbonized green algae	20	83.0	1.35	15 d	1	[197]
	Polypyrrole/filter paper	3.5		1.04	70h	1	[198]
	Biomass‐ hydrogel	25		1.77	7 12 h	1	[136]
	Polyethylene glycol / graphene oxide	20	88.0	1.32	15 h	1	[199]
	MoS_2_/cotton	7.5	84.5	1.38	120 h	1	[200]
	T‐ shaped PPy/PVA		75.0	2.03	60h	1	[201]
Siphon Cappilary effect	Cone‐shaped 3D evaporator	3.5		1.71	126h	1	[202]
	PDA‐PEI@ASA	20		2.55	168h	1	[62]
	Hollow fiber membranes	20	229.1	3.65		1	[140]
Zwitterionic Effect	Sunflower pith	20	98.8	1,90	50h	1	[66]
	PIC/PAni hydrogel	3.5	96.3	2.79	10h	1	[149]
	PU/CNT fiber	20	93.5	2.20	2h	1	[148]

Summary of the Table 4

Additionally, as shown in Table 5, the six salt blocking strategies are compared using the same criteria in terms of salt rejection position, dominating driving force, water‐supply pathway, tolerated salinity, stability duration, salt‐harvesting capability, and heat‐loss penalty.

**TABLE 5:** The summary of six types of salt‐blocking strategy.

Strategy	Salt rejection location	Driving force	Water‐supply pathway	Salinity tolerance	Stability	Salt harvesting	Heat loss	Refs.
Hydrophobic effect	Interface or surface	Interfacial energy barrier	Through hydrophobic layer	Moderate	High	Poor	Low	[203, 204, 205, 206]
Unidirectional brine transport	Bulk to away from interface	‐Capillary‐Pressure–Gradient	Asymmetric channels	High	High	Limited	Medium	[207, 208]
Dissolution migration	Within bulk water	Concentration gradient	Diffusive transport	Moderate to high	Medium	Poor	higher	[209, 210]
Edge crystallization	At edges	Evaporation‐induced flow	Lateral flow	High	High	Excellent	Medium	[211, 212, 213]
Siphon capillary effect	Remote reservoir	Capillary siphoning	Continuous liquid bridge	High	High	Limited	Higher	[214, 215, 216]
Zwitterionic effect	Interface	Hydration layer or electrostatic	Through hydrophilic layer	Moderate	High	Poor	Low	[217, 218, 219, 220]

Summary from Table 5 Edge crystallization and unidirectional brine transport are very stable and tolerant to high salinity, effective for long‐time operation, and edge crystallization has excellent salt harvesting capability. The siphon capillary effect has a high salinity tolerance, but a higher heat loss. Hydrophobic and zwitterionic effects, however, originate from interfacial phenomena that allow for lower heat loss and high stability, but are limited to salt harvesting and are only moderately tolerant of salinity. Dissolution migration gives moderate to high tolerance by diffusive transport, but is not efficient for salt removal.

## 3 Conclusion

Over the past decade, due to the dedication of numerous researchers as well as experts from different disciplines, significant improvements have been achieved in desalination and fresh water production. Therefore, timely reports on the progress of anti‐salt fouling strategies are very crucial. In this review, we discussed advanced strategies for salt scaling prevention technology in hypersaline water, including the hydrophobic effect, unidirectional brine transportation, zwitterionic effect, edge crystallization, siphon capillary effect, and migration of salts by dissolution were extensively explored. Finally, we recommended our perspectives from a scientific point of view to shape the direction of research and promote its practical implementation. For further research we recommend that multiscale porous structures integrating edge crystallization and unidirectional transport to balance water supply, evaporation, and salt removal.

## 4 The Challenges and Outlook

Despite remarkable progress in solar desalination, some challenges still exist. The following points address the current challenges and future prospects of solar desalination.

**FIGURE 8:**
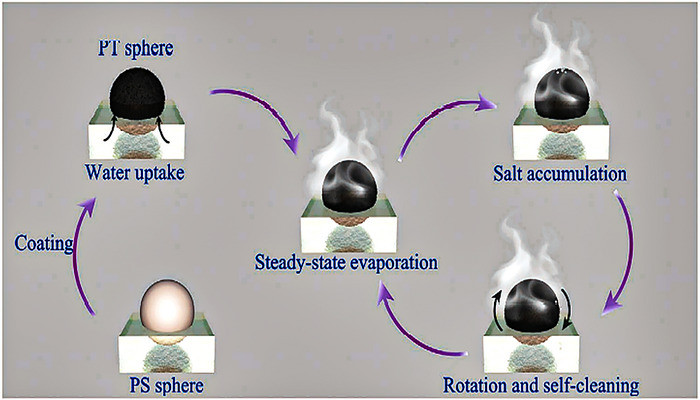
Scheme illustration of photothermal sphere fabrication and salt‐resisting mechanism, reproduced with permission [223]. Copyright 2021, Wiley.

- The durability of evaporation is a redoubtable challenge due to the unpreventable accumulation of salt in the evaporator, particularly in hypersaline water desalination. The scaling of salts on the evaporator surface is directly related to the salinity of the feed. These salts block the absorption of light, hamper vapor escape, significantly decline the water evaporation rate, and finally shorten the lifetime of photothermal evaporators. Thus, before commercializing, scholars should test the effectiveness of their design durability at high salinity and high power density rather than under one sun.
- On the laboratory scale, extremely high photothermal conversion efficiency has been achieved even beyond theoretical limits (i.e., greater than 100%). Researchers use different formulas to calculate the photothermal conversion efficiency. At the current status, there is no standardized formula to compare different efficacies. In the future, it should be set.
- Most researchers utilized simulated seawater (pure NaCl) in a beaker, which is quite different from actual seawater, which may contain bacteria, fatty acids, proteins, oil, and different heavy metal ions that accelerate the scaling of the salts and other contaminants on the surface of photothermal conversion material. Except for a few studies, systematic exploration using real seawater is still lacking.
- Along with seawater desalination design, synergistic application is very important. For instance, by using salt concentration differences to generate electricity, using that electricity further for wave detection on the seashore, and also by taking advantage of the surface charge of the evaporator during desalination, it is also possible to selectively extract precious elements like gold. This multifunctional application aims to acquire sustainable solutions for different resources.
- In addition to the six salt scaling prevention strategies mentioned, new designs for seawater desalination should be explored in the near future. Recently, promising solar desalination has also been developed [221, 222]. For instance, Wu et al. [223]. Specifically designed a self‐rotating new evaporator with two evaporation zones (i.e., low‐temperature and high‐temperature zones). The designed evaporator is sensitive to salt accumulation and induces a quick rotation of the evaporator. After rotation, the deposited salt was dissolved back into the seawater, and the surface of evaporation was periodically refreshed. Additionally, the low‐temperature evaporation zone eliminated heat loss into bulk seawater and gained extra energy from the environment. This design is properly working in hypersaline water with a high 2.6 kg m^−2^ h^−1^ rate of water evaporation. Figure 8 illustrates the synthesis and working mechanism of the evaporator.

## Conflicts of Interest

The authors declare no conflicts of interest.

## Data Availability

The data that support the findings of this study are available from the corresponding author upon reasonable request.
